# Correlation Between the *TAS2R38* Genetic Polymorphism, PROP Perception and Eating Habits: A Cross-Sectional Molecular Epidemiological Study

**DOI:** 10.3390/epidemiologia7050131

**Published:** 2026-09-17

**Authors:** Tommaso Rondini, Lisa Parri, Maya Petricciuolo, Cristina Fatigoni, Milena Villarini, Ermanno Federici, Massimo Moretti, Mattia Acito

**Affiliations:** 1 Department of Pharmaceutical Sciences, University of Perugia, Via del Giochetto, 06122 Perugia, Italy; tommaso.rondini@outlook.it (T.R.); cristina.fatigoni@unipg.it (C.F.); milena.villarini@unipg.it (M.V.); massimo.moretti@unipg.it (M.M.); 2 Independent Researcher, 06024 Gubbio, Italy; lisaparri@hotmail.it; 3 Department of Chemistry, Biology, and Biotechnology, University of Perugia, Via del Giochetto, 06122 Perugia, Italy; maya.petricciuolo@unipg.it (M.P.); ermanno.federici@unipg.it (E.F.); 4 School of Pharmacy, University of Camerino, Via Madonna delle Carceri 9, 62032 Camerino, Italy

**Keywords:** bitter taste, PROP, *TAS2R38*, food preferences, Mediterranean diet, human health

## Abstract

**Background/Objectives**: Genetic variation in bitter taste receptors, particularly *TAS2R38*, influences sensitivity to bitter compounds such as 6-n-propylthiouracil (PROP). However, its influence on food preferences and dietary habits remains unclear. This study investigated the relationship between *TAS2R38* rs713598 polymorphism, a variant in strong linkage disequilibrium with other common variants and therefore served as a proxy for TAS2R38 bitter taste-related genetic variation, PROP responsiveness, food preferences, and adherence to the Mediterranean diet. **Methods**: A cross-sectional molecular epidemiological study was conducted on 200 Italian adults. Participants completed questionnaires assessing food preferences, Mediterranean diet adherence (energy-restricted Mediterranean Diet Adherence Screener; er-MEDAS), and physical activity (IPAQ). Bitter taste perception was evaluated using the PROP test and a Labelled Magnitude Scale. Saliva samples were collected for *TAS2R38* rs713598 genotyping by qPCR. Associations were analysed using Spearman’s correlation and generalised linear models. **Results**: Genotyping was completed for 176 participants. A significant positive correlation was found between rs713598 genotype and PROP sensitivity (ρ = 0.404, *p* < 0.01). Women showed higher PROP responsiveness than men. No significant associations were observed between PROP responsiveness or *TAS2R38* genotype and either food preferences or Mediterranean diet adherence. Older age was associated with greater preference for bitter foods (*p* = 0.008), whereas higher physical activity and non-smoking status were associated with greater adherence to the Mediterranean diet (*p* = 0.020 and *p* = 0.024, respectively). **Conclusions**: *TAS2R38* polymorphism influenced bitter taste perception but showed limited relevance for food preferences and dietary habits. Lifestyle factors appeared more influential in shaping dietary behaviour.

## 1. Introduction

Food preferences are shaped by genetic, cultural, social, psychological, and socioeconomic factors. These habits are often deeply rooted in individuals, making them difficult to change [1]. Moreover, taste perception depends on the gustatory system, which is crucial in food choices. This system protects us from potentially harmful substances and helps us to ingest vital nutrients [2]. In particular, the perception of bitterness serves a twofold function: it can be an attractive stimulus for consuming certain products or a warning signal. In fact, intense bitterness is often indicative of potentially harmful substances, and for this reason, such foods are generally avoided [3].

Unlike the other basic taste modalities, bitter taste perception is characterised by remarkable genetic variability. Indeed, in the human genome, there are 25 functional genes and 11 pseudo-genes belonging to the TAS2R family of bitter taste receptors located on chromosomes 4p, 7p, and 12p, and about 550 molecules capable of binding to these receptors have been identified. It has been suggested that the polymorphic variants of these genes are responsible for the variability observed in different human phenotypes and lifestyle habits such as alcohol consumption and nicotine addiction, as well as in plasma antioxidant status [4,5,6,7,8,9].

The *TAS2R38* gene is the most studied in relation to the perception of bitterness [4]. This gene exhibits two predominant haplotypes, PAV (Proline–Alanine–Valine) and AVI (Alanine–Valine–Isoleucine), which differ at three amino acid positions (A49P, A262V, V296I). These variants are generally associated with the “taster” (PAV) and “non-taster” (AVI) phenotypes. The SNP rs713598, corresponding to the A49P substitution, is in strong linkage disequilibrium with the other two polymorphisms and can therefore be used as a proxy marker for the PAV/AVI haplotypic variation, although it does not by itself define the complete haplotype [8,9,10].

*TAS2R38* has been widely studied for its role in taste perception and food preference, particularly in early life, and has also been investigated as a genetic marker for susceptibility to behaviours and conditions such as smoking and obesity [4,6,7]. Moreover, since the receptor encoded by this gene binds compounds containing the thiourea group (N-C=S) associated with bitter taste, *TAS2R38* polymorphisms could influence the consumption of food containing this ligand, such as vegetables belonging to the *Brassicaceae* family [8,9]. Consequently, an indirect effect of the ability to taste bitter compounds associated with these polymorphisms could be the avoidance of these products, which, at the same time, present several nutraceutical compounds with proven health properties [10]. In addition to these vegetables being largely consumed in a Mediterranean diet, as well as other foods related to bitter taste (e.g., extra virgin olive oil, tea, coffee, wine, etc.), it could be plausible that polymorphisms of this gene could influence adherence to such a healthy dietary pattern. Nevertheless, the association between *TAS2R38* genetic variability and adherence to the Mediterranean dietary pattern remains largely unexplored, warranting further investigation.

Overall, regarding food choice, recent evidence suggests that the influence of bitter taste sensitivity on dietary behaviour is generally modest and that the relationship between *TAS2R38*, food preferences, and dietary intake remains inconsistent across different populations and study designs [11,12,13]. Therefore, further molecular epidemiological studies are needed to clarify the relative contribution of genetic and lifestyle factors to dietary behaviour.

In light of the above, this study aims to investigate whether there is a correlation between the consumption of cruciferous vegetables and other bitter foods, or adherence to the Mediterranean diet—a well-studied dietary pattern known for its beneficial effects on health—and individual sensitivity to bitterness, as assessed through the 6-n-propylthiouracil (PROP) test, a recognised oral marker of food preferences [11,12,13,14,15], as well as the detection of the *TAS2R38* genetic polymorphism from participants’ saliva.

## 2. Materials and Methods

### 2.1. Study Design and Sampling Strategy

This study is a cross-sectional descriptive molecular epidemiology investigation. Participants were recruited through information stands set up in companies, gyms, universities, and shops across the city of Perugia (Italy).

The sample size was determined according to the recommendations of Green [14] for multivariable regression analysis (N ≥ 50 + 8*m*), where *m* represents the number of predictor variables. Since seven independent variables (i.e., age, sex, BMI, smoking, physical activity, PROP score, *TAS2R38* genotype) were included in the model, a minimum sample size of 106 participants was required. To ensure that this minimum sample size was achieved after data cleaning and laboratory analyses, the recruitment target was increased to 200 participants. This strategy accounted for potential participant attrition, including incomplete questionnaires, refusal or failure to provide saliva samples, and unsuccessful or indeterminate *TAS2R38* genotyping.

At each site, the study’s aims and procedures were first explained, after which individuals were invited to participate. Those who agreed were asked to provide written informed consent. After completing a questionnaire to obtain anthropometric, socio-economic, and lifestyle data (e.g., eating habits and physical activity), participants were asked to perform the PROP test to rate their perception of bitter taste. Moreover, saliva samples were collected using a dedicated kit, subsequently coded to ensure anonymity. Finally, DNA was extracted from the saliva and analysed to investigate the presence of polymorphisms in the bitter taste receptor gene *TAS2R38*.

Participation in the study was voluntary, and no incentives were offered.

Individuals were eligible if they were aged 18 years or older, provided written informed consent, completed the study questionnaires, underwent the PROP taste test, and agreed to provide a saliva sample for *TAS2R38* genotyping. To standardise PROP responsiveness assessment and saliva collection, participants were excluded if they had eaten or consumed beverages other than water shortly before the examination. Individuals were also excluded if they had consumed coffee, alcohol, smoked, chewed gum, used mouthwash, or brushed their teeth within 30 min before testing. These measures were adopted to reduce potential sources of bias related to taste perception and salivary sample quality.

Participants who declined participation, failed to complete the questionnaires or PROP test, or provided saliva samples of insufficient quality for DNA analysis were excluded from the corresponding analyses.

### 2.2. Socio-Economic and Anthropometric Data Collection

The first part of the questionnaire consisted of standard questions such as education, residence, etc.

Participants were also asked to specify their height and weight to calculate their body mass index (BMI), and whether they smoked and how many cigarettes (Table 1).

### 2.3. Food Preference Questionnaire

Participants were given a Food Preference Questionnaire [15], adapted to the eating habits of the Italian population. The questionnaire contained 23 foods (please see Supplementary Materials, Figure S1) associated with bitter taste, and a 5-point scale (1 = “I hate it” to 5 = “I love it”) as previously described for food preference assessment [15,16]. Participants were asked not to discuss their food preferences while completing the questionnaire.

Many of the products are vegetables from the *Brassicaceae* family, as they contain glucosinolates and goitrin, which are characterised by the presence of the thiourea chemical group, one of the main ligands of the TAS2R38 bitter taste receptor [17,18,19,20,21]. *Brassicaceae* vegetables also provide vitamins, plant-based fibres, and bioactive compounds such as isothiocyanates, which have been associated with detoxification, anti-inflammatory, and anticancer pathways [10]. Their potential health benefits may depend on consumption frequency and amount, as well as on the enzymatic process involved in isothiocyanate formation [22]. Consequently, it could be important to understand whether people consume these products also as a function of their bitter perception.

Actually, rather than evaluating TAS2R38-related responses to this specific botanical food group, the questionnaire was conceived to assess general preferences for bitter-tasting foods commonly consumed within the Italian and Mediterranean dietary context. Therefore, the 23 food and beverage items were selected to encompass a broad range of products characterised by naturally occurring bitter or potentially bitter sensory components, rather than being limited exclusively to *Brassicaceae* vegetables. The selection included vegetables and other plant-based foods, herbs and spices, as well as commonly consumed beverages and foods such as coffee, tea, red wine, beer, and dark chocolate. Finally, a Food Preference Score was calculated by summing the scores assigned by the participants to each food item included in the questionnaire. Hence, the higher the score, the more people liked these bitter foods.

### 2.4. Mediterranean Diet Adherence

To get a general overview of eating habits, Mediterranean diet adherence was assessed through a validated questionnaire (i.e., 17-item er-MEDAS; please see Table 2) [23] with minor modifications, considering the 14-item er-MEDAS questionnaire [24]. Finally, the questionnaire included 14 items on food consumption and 2 on eating behaviours. Compliance with each of the 16 items was scored with 1 point. Therefore, the total er-MEDAS score range was 0 to 16, with 0 meaning null adherence and 16 meaning maximum adherence.

As a consequence, according to Bouzas et al. [25], people were classified into three Mediterranean diet adherence classes: 0 to 7 (low adherence), 8 to 10 (moderate adherence), and 11 to 16 (high adherence). The frequency of the consumption of different Mediterranean products leading to a score of 1 point on the er-MEDAS and the detailed questionnaire with appropriate edits are displayed in Table 2 of the Results and Discussion section.

### 2.5. PROP Test

Sensitivity to the bitter compound 6-n-propylthiouracil (PROP) has been widely adopted as a phenotypic biomarker of interindividual variability in bitter taste perception, mainly related to polymorphisms in the *TAS2R38* gene [17,18,19].

Similar to phenylthiocarbamide (PTC; [26]), people can be defined as bitter “tasters” and “non-tasters” based on their different sensitivity to PROP. Although both compounds display similar properties, PROP is generally used for taste blindness since it lacks the typical sulphurous odour of PTC and does not exhibit the toxicity that is associated with the latter [27].

The PROP tests (purchased by Bartovation LLC., New York, NY, USA) consisted of sterile filter paper discs (~2.5 cm in diameter) soaked with 100 µL of 5 mM PROP solution. Participants were asked to place the paper disc on their tongue, moisten it with saliva, and mimic chewing for at least 10 s. Subsequently, participants received a copy of the Labelled Magnitude Scale (LMS) [28] with minor modifications. The scale instructions ask the participant to think of “any kind of strongest sensation imaginable” and rate it as 10 on the scale [29]; values ranged from 0 = “barely detectable” to 10 (please see Supplementary Figure S2).

### 2.6. International Physical Activity Questionnaire (IPAQ)

Since physical activity is closely associated with dietary behaviours and healthier eating patterns [30], assessment of the participants’ lifestyle habits was considered essential to control for potential confounding factors in the relationship between bitter taste perception and food choices. Therefore, the last part of the questionnaire focused on health-related physical activity evaluation through the validated International Physical Activity Questionnaire (IPAQ) [31].

This consists of nine questions, distinguishing vigorous (requires a high level of physical effort and forces you to breathe faster than normal) and moderate (requires moderate physical effort and breathing at a rate that is only moderately higher than normal) physical activity, walking, and sitting activities.

The questions are meant to define how much time people have spent doing physical activity in the last 7 days. They include questions about activities they do at school, moving from one place to another, and finally, during their free time.

The Metabolic Equivalent of Tasks (MET), which is defined as “the amount of oxygen consumed while sitting at rest, equals 3.5 mL O_2_ per kg of body weight per minute” [32], was calculated for each type of physical activity (vigorous, moderate and walking). Finally, a total MET score was obtained to distinguish active, sufficiently active, and inactive people, as follows:

− total MET lower than 700 means an inactive person;
− total MET between 700 and 2519 means a sufficiently active person;
− total MET higher than 2520 means an active or very active person.

### 2.7. DNA Saliva Sample Collection and Isolation

Saliva was collected through a proper collection kit (Zeesan Biotech Co., Ltd., Xiamen, China), which consisted of a collection funnel attached to a lid and a collection tube prefilled with preservation solution.

Participants were asked not to rinse their mouths to remove food particles. Samples were stored at room temperature, and subsequently, the DNA was isolated with a column-based isolation kit from Norgen Biotek Corporation (Thorold, ON, Canada) according to the following procedure.

Briefly, for lysate preparation, 250 µL of collected saliva was transferred into a sterile microcentrifuge tube to which 250 µL of lysis buffer and 20 µL of proteinase K were added. After vortexing, the samples were incubated at 55 °C for 10 min. Subsequently, 200 µL of binding buffer was added, followed by a further 5-min incubation at 55 °C. Then, 700 µL of isopropanol was introduced and briefly mixed. Saliva DNA isolation was performed by assembling the column with a collection tube and applying the lysate. Centrifugation at 6000 RPM (~3800× *g*) using a fixed-rotor centrifuge enabled passage of the lysate through the silica membrane, where DNA was retained. After washing, two further centrifugation steps were carried out (first at 8000 RPM and then at a maximum speed of 15,000 RPM). Finally, DNA was eluted from the column membrane using the elution buffer and collected in a clean collection tube. DNA samples were stored at −20 °C.

### 2.8. TAS2R38 Genetic Polymorphism Detection

The SNP rs713598 in the *TAS2R38* gene of the DNA samples obtained from saliva collection was analysed through real-time PCR (TaqMan), using the relevant kit purchased from Clonit S.r.l. (Milan, Italy).

The kit used contained two conventional primers, expressly designed to amplify the sequence surrounding rs713598, and two single-stranded probes. The latter are complementary either to the G or the C variant and are marked at the 5′-end with a unique reporter fluorescent molecule (one probe with VIC fluorophore, and one with FAM). The Taq polymerase degrades the probes attached to the amplicons, releasing reporters and allowing the detection of their signals, and the presence of both alleles can be assessed depending on the fluorescence emission. Since only the rs713598 SNP was genotyped in the present study, the resulting genotypes were reported as P/P, P/A, and A/A, according to the amino acids encoded.

DNA concentration was initially quantified by fluorometry using a Quantus™ Fluorometer (Promega Corporation, Madison, WI, USA) with the QuantiFluor dsDNA System. DNA samples were subsequently diluted to a final concentration of 8 ng/µL for genotyping. The complete master mix was then prepared (reaction volume: 12.5 µL of TAS-RQ Master Mix, 1.2 µL of proper probe solution, and 8.8 µL of double-distilled water) and aliquoted into 22.5 µL in a PCR tube strip. Subsequently, 2.5 µL of each sample was added, and the qPCR run was performed using the StepOne Real-Time PCR System (Thermo Fisher Scientific Inc., Waltham, MA, USA).

Proper positive (GG, CC, and GC) and negative (double-distilled water) controls were performed.

### 2.9. Statistical Analysis

The database setup and all statistical analyses were performed using Excel (Microsoft Office 365 suite) and SPSS for Windows version 20.0 (SPSS Inc., Chicago, IL, USA).

Results were expressed as the mean ± standard deviation (SD) for age, er-MEDAS score, food preferences score, PROP test score, and MET/min/week. Otherwise, frequencies were shown as numbers and percentages. Considering the obtained genotype analysis, the Hardy–Weinberg and χ^2^ tests were performed to verify the genetic consistency of the sample. Avoiding normal distribution of the data, the strength and direction of the monotonic relationship between the *TAS2R38* genotype and PROP score was assessed through the nonparametric Spearman’s rho test (ρ). The generalised linear models analysed the dependence of bitter product consumption and Mediterranean diet adherence on one or more independent variables (e.g., age, BMI, smoking habit, PROP, etc.).

The level of statistical significance was set at *p* < 0.05.

### 2.10. AI Use

During the preparation of this manuscript/study, ChatGPT (OpenAI, GPT-5.5, San Francisco, CA, USA) was used as a writing assistant for language and grammar. We have reviewed and edited the output and take full responsibility for the content of this publication.

## 3. Results

### 3.1. Socio-Demographic and Lifestyle Characteristics

Among the 200 enrolled participants in the study, most of them were female (67.5%), while 32.5% were male. Regarding age distribution, 64.0% of participants were younger than 30 years, whereas 36.0% were aged 30 years or older.

Regarding lifestyle traits (Table 1), 26.1% of the sample consisted of smokers, with an average consumption of 7.41 ± 5.78 cigarettes per day. BMI analysis showed that 76.5% of participants were classified as normal weight, 15.5% as overweight, 3.5% as obese, and 4.5% as underweight. According to the IPAQ classifications, 45.0% of participants were physically inactive, 36.5% sufficiently active, and 18.5% active.

### 3.2. Mediterranean Diet Adherence and Food Preferences

Sampled population eating habits are summarised in Table 2. Most participants reported using extra-virgin olive oil as their primary cooking fat (97.0%).

Compliance with Mediterranean dietary recommendations varied considerably among food categories. Daily vegetable intake recommendations were met by 59.0% of participants, whereas only 24.5% consumed at least three portions of fruit daily.

Only 16.0% of participants consumed fish and seafood in accordance with the Mediterranean diet recommendations, while 28.0% and 35.5% met the recommendations for legume and nut consumption, respectively. Even though the preference between red meat and white meat was equally distributed, with 48.5% preferring the latter, only 23.0% of people consumed one or fewer servings (one portion = 100–150 g) of red meat. Moreover, although 65.5% of the participants drank sugar-sweetened and/or carbonated beverages more than 1 time a week, the majority (60.0%) did not routinely add sugar to drinks such as coffee or tea, and 48.0% tended to consume pastry foods less than three times a week. Weekly, 37.5% of individuals consumed fewer than three servings (one portion = 75 g) of refined-flour bread, rice, and pasta, while a nearly identical proportion (36.5%) consumed the whole-wheat equivalents. Regarding alcohol consumption (wine, beer, and strong drinks), only 3.0% of people met the specified servings.

Overall, 69.0% of participants showed low adherence to the Mediterranean diet, while 31.0% demonstrated moderate/high adherence (Table 3).

**Figure 1:**
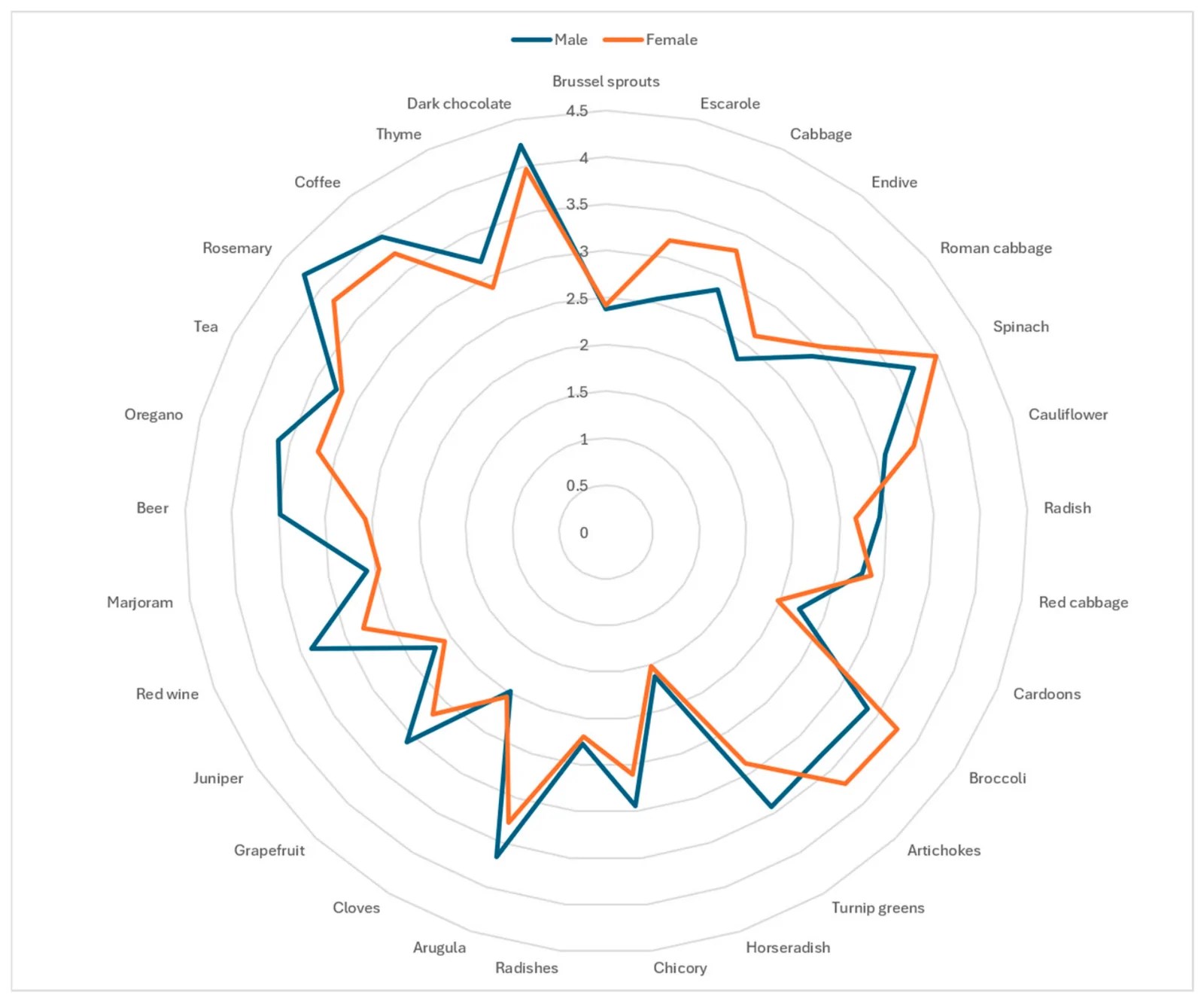
Male and female food preferences.

Based on the participants’ food preferences (Table 3 and Figure 1), the mean score of the sampled population was 85.56 ± 20.78. Dark chocolate, rosemary, spinach, and coffee received the highest preference scores, whereas horseradish, cloves, cardoons, and juniper received the lowest.

### 3.3. PROP Perception and TAS2R38 Genotyping

Among the 176 successfully genotyped participants, 21.0% were classified as super-tasters (P/P), 50.0% as medium tasters (P/A), and 29.0% as non-tasters (A/A). The indeterminability of some samples occurred as DNA extracted from saliva may contain variable amounts of non-human DNA (bacterial/fungal) and inhibitory compounds (mucins, polyphenols, etc.) [33,34]. Hardy–Weinberg equilibrium analysis (Table 4) showed no significant deviation between the observed and expected genotype frequencies. In addition, to assess potential selection bias related to unsuccessful genotyping, participants with successful genotyping (*n* = 176) were compared with those whose genotyping results were indeterminate (*n* = 24). No statistically significant differences were observed between the two groups (*p* > 0.05).

The PROP test (Table 4) highlighted a clear difference between genders in the perception of bitter taste. Women were more sensitive than men: 17% of women classified the perception as “Very strong—The strongest imaginable”, compared to only 6.2% of men. Sixty percent of men (only 47.4% of women) classified the bitterness as “Barely detectable—Weak”. The average PROP scores, displayed in Table 4, further confirmed this difference: women rated a score of 2.51 ± 2.51, clearly higher than men (1.77 ± 2.12), indicating greater sensitivity to bitter taste among female participants.

**Figure 2:**
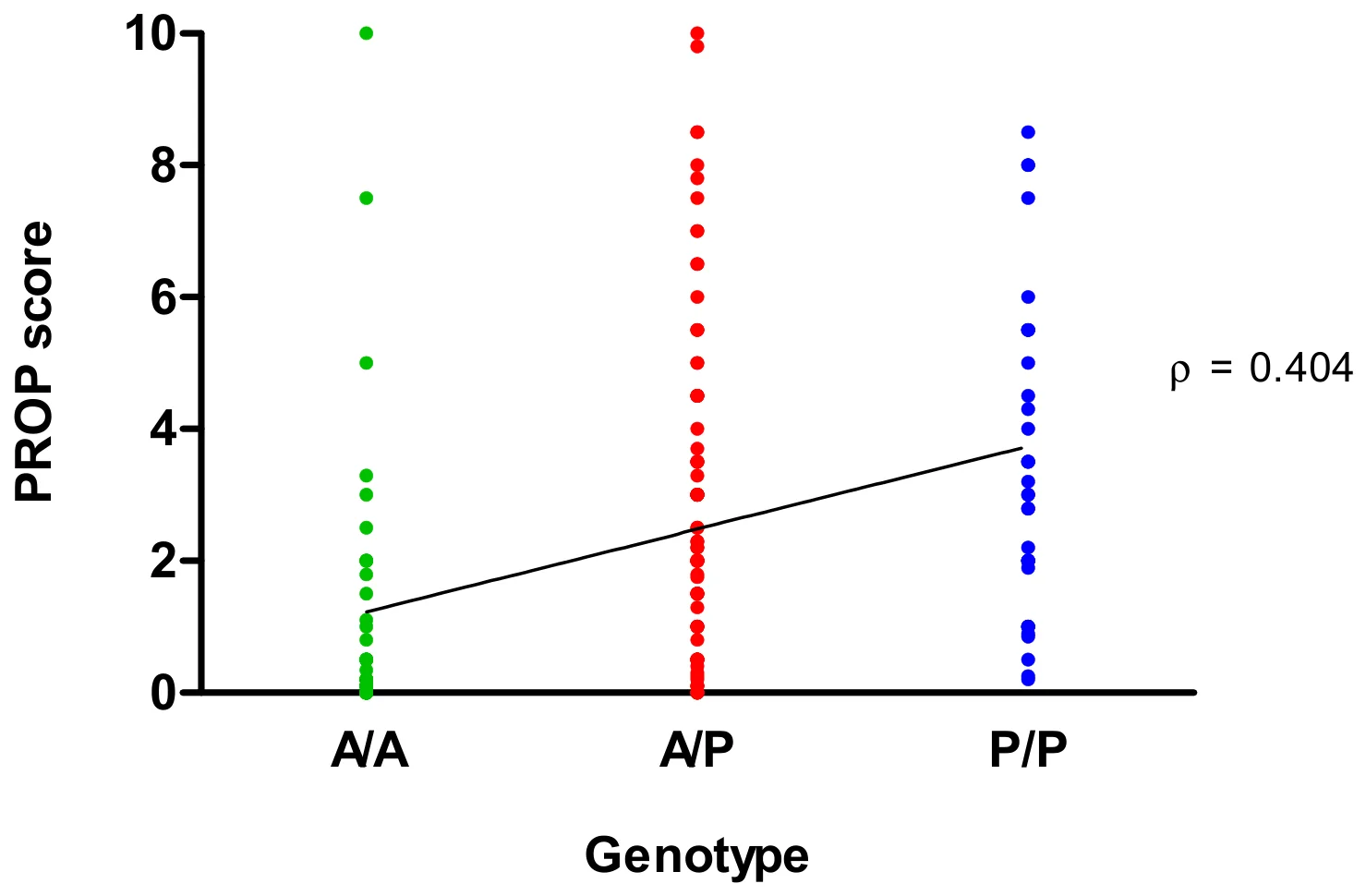
Correlation between PROP score and rs713598 *TAS2R38* SNP genotypes (A/A = “non-taster”; P/A = “medium taster”; P/P = “super-taster”); Spearman’s ρ = 0.404, *p* < 0.01. The analysis was performed among participants with available genotyping data (*n* = 176).

Finally, the Spearman’s rank correlation analysis (Figure 2) demonstrated a moderate positive correlation between *TAS2R38* genotype and PROP score (ρ = 0.404; *p* < 0.01).

### 3.4. Association Between Lifestyle Factors, Food Preferences, and Mediterranean Diet Adherence

The generalised linear model (GLM) analysis (please see Supplementary Tables S1 and S2) was carried out to verify whether there were associations between food preferences/Mediterranean diet adherence, selected as dependent variables, and independent variables classified as modifiable (i.e., smoking, physical activity and BMI) or non-modifiable (i.e., sex, age, *TAS2R38* genotype with SNP rs713598 and PROP bitter perception).

Only age was significantly associated with food preference scores (*p* = 0.008), whereas no statistically significant associations were observed between food preferences and PROP sensitivity or *TAS2R38* genotype. Mediterranean diet adherence was negatively associated with smoking habit (*p* = 0.024) and positively associated with physical activity levels (*p* = 0.020). No significant associations emerged between Mediterranean diet adherence and PROP sensitivity or *TAS2R38* genotype.

## 4. Discussion

The present study explored the relationship between bitter taste perception, genetic variability in the TAS2R38 bitter taste receptor, food preference, and adherence to the Mediterranean diet.

Attention was given to bitter-tasting foods, including several vegetables belonging to the *Brassicaceae* family. These vegetables contain glucosinolates and goitrin, compounds characterised by the presence of a thiourea chemical group, which represents one of the main ligand structures recognised by the TAS2R38 bitter taste receptor [17,21]. Beyond their sensory characteristics, *Brassicaceae* vegetables are relevant from a nutritional perspective because they provide vitamins, plant-based fibre, and bioactive compounds with potentially beneficial health effects, depending on intake frequency and on the enzymatic processes involved in isothiocyanate formation [17]. These latter potentially contribute to detoxification, anti-inflammatory, and anticancer mechanisms [17,18,19,20,22]. Therefore, understanding whether individual differences in bitter taste perception may influence the consumption of these foods is of nutritional interest.

More broadly, the Mediterranean diet is characterised by a high consumption of plant-based foods, including *Brassicaceae* vegetables [35]. Adherence to this dietary pattern reflects a complex interplay of sensory, cultural, behavioural, and lifestyle factors, raising the question of whether genetic differences in bitter taste perception may contribute to individual dietary choices.

Results confirmed a statistically significant association between PROP sensitivity and *TAS2R38* rs713598 genotype, supporting the role of this polymorphism as a biomarker of bitter taste perception. These findings are consistent with previous literature [24,25,26,27] reporting that PAV/PAV individuals generally exhibit higher sensitivity to PROP, whereas AVI/AVI carriers tend to show reduced bitter taste perception.

Nevertheless, although PROP responsiveness and *TAS2R38* genotype were strongly associated with each other, neither variable showed significant associations with food preferences or adherence to the Mediterranean diet. These findings suggest that the relationship between bitter taste genetics and real-life dietary behaviour may be weaker and more complex than expected. The literature on this subject is highly heterogeneous. For instance, some studies have shown that differences in PROP sensitivity have been associated with variations in the acceptance and intake of fruits and vegetables [3,24,25,26,27], even though most focused on hedonic ratings, food choice, or sensory perceptions regarding bitter foods, but not self-reported food preferences. On the contrary, numerous studies highlighted the absence of a direct and clear correlation, as various confounding variables may influence the results [11,24,25,26,27,28,29].

Multiple factors, including culture and the learning process, shape food preferences, and consequently, the dietary regimen. In fact, although *TAS2R38* genetic polymorphisms can influence the perception of some bitter vegetables, this effect alone is probably insufficient to modify a composite dietary pattern such as the Mediterranean diet, which depends on many cultural choices and habits [36]. As a demonstration of this, the existing literature reports findings concerning other eating behaviours, such as overall food intake, which likewise remain inconclusive [25,26,27,28].

While our genetics regarding PROP sensitivity may predispose us to perceive certain tastes as unpleasant, positive experiences and social learning can help us develop an “acquired taste” for initially aversive flavours, such as bitterness [15,37,38]. Additionally, exposure to bitter foods since childhood, such as cruciferous vegetables, and parental educational approaches can promote long-term acceptance of such foods [15]. Therefore, rejection might be related to other factors beyond bitter taste, such as visual appearance, texture, or cultural or social barriers [39]. Another possible factor that could explain the results is that cultural habits, cooking techniques and seasonings can attenuate the aversion to bitterness; therefore, the innate perception does not necessarily translate into avoidance (e.g., fats, salt, sugar, etc.) [9,40].

In addition, it should be noted that taste perception is a complex, polygenic trait mediated by the interaction of numerous receptors and genes within the TAS2R family (approximately 25 in mammals), each contributing differently to individual sensitivity to bitter compounds. Consequently, the analysis of a single polymorphism, such as rs713598 in the *TAS2R38* gene, provides only a partial representation of the genetic determinants underlying bitter taste perception and their potential influence on dietary behaviour [29,30,31].

A greater inclination toward bitter-tasting food consumption was observed as age progressed, despite a potential decline in taste sensitivity, such as that caused by a reduction in taste receptors [41]. This phenomenon is linked to a combination of long-standing habits [41], an increased focus on health [42], and the effect of repeated exposure to foods [43]. Such exposure gradually helps overcome initial aversions, promoting broader acceptance and transforming these foods into familiar and appreciated choices [44,45]. Consequently, with advancing age, the role of genetics in taste perception diminishes, giving way to cultural, experiential, and behavioural factors [37,41].

Interestingly, the data obtained suggest that smokers tended to consume the bitter foods listed in the questionnaire less frequently than non-smokers, although this result was not statistically significant. Even so, considering that most of the foods included in the questionnaire are vegetables and that plant-based products form the basis of the Mediterranean food pyramid [46], the association appears to be supported by the results of the correlation analysis between unmodified factors and adherence to the Mediterranean diet, which showed, in line with other studies [47,48], that smokers adhere significantly less to the Mediterranean pattern (*p* = 0.024). Regarding taste perception, some studies from the literature did not identify a significant effect of cigarette smoking [16,49]. Conversely, other research suggested that smoking may reduce taste perceptions, while quitting smoking could help restore them [50]. For instance, Tepper et al. showed that after just two weeks of smoking cessation, individuals experienced an increase in taste intensity perception as measured by the PROP test [51].

The Mediterranean diet adherence also appeared to be influenced by physical activity. In particular, the more people were physically active (high MET/min/week), the more they adhered to a healthy eating style like the Mediterranean one (*p* = 0.020). This association is consistent with the rationale for including physical activity in the present study, as physical activity is closely associated with dietary behaviours and healthier eating patterns and may therefore represent an important lifestyle-related confounding factor when examining the relationship between bitter taste perception and food choices [30]. A similar trend was observed when analysing bitter vegetables and food preferences, though without a statistically significant result. In fact, having a healthier lifestyle, such as practising regular physical activity, may help decrease the attraction to high-fat foods, instead promoting a greater preference for lighter, lower-calorie options (such as vegetables), all accompanied by an improvement in the ability to regulate appetite [52]. Furthermore, other studies highlighted that individuals who engage in more physical activity were more focused on foods’ nutritional and health benefits rather than taste itself [53,54,55].

Collectively, these findings suggest that public health interventions aimed at enhancing population health outcomes should pivot their focus. Namely, rather than prioritising genotype-based nutritional strategies, initiatives should probably concentrate on comprehensive behavioural education and the systematic promotion of repeated exposure to diverse flavours.

By emphasising the critical role of social learning, positive experiential outcomes, and varied culinary techniques in mitigating innate sensory aversions, these strategies can effectively facilitate the development of an “acquired taste” for nutritionally dense but bitter-tasting vegetables, such as the *Brassicaceae* family. Ultimately, focusing on modifiable lifestyle factors—such as increasing physical activity and addressing smoking habits—appears to be a more robust approach for shaping healthy, long-term dietary behaviours than merely focusing on unchangeable genetic predispositions.

### Strengths and Limitations

This study has several strengths, including the integration of genetic, phenotypic, dietary, and lifestyle assessments within the same study population. In particular, the combined evaluation of the *TAS2R38* rs713598 polymorphism and PROP responsiveness allowed the genetic component of bitter taste perception to be considered alongside self-reported food preferences and adherence to the Mediterranean diet. The assessment of physical activity and smoking habits also provided additional information on relevant lifestyle factors potentially contributing to dietary behaviour.

Nevertheless, some aspects should be considered when interpreting the findings. The genetic analysis focused on a single polymorphism in the *TAS2R38* gene. Although rs713598 has strong linkage disequilibrium with the other SNPs and displayed a significant association with PROP responsiveness, bitter taste perception is a complex polygenic trait involving multiple taste receptors and genetic variants. The availability of additional genetic markers could therefore provide a more comprehensive characterisation of the genetic contribution to taste perception and dietary behaviour. This aspect represents an opportunity for future studies to extend the present findings through a broader genetic characterisation while maintaining a multidisciplinary approach that integrates genetic, sensory, behavioural, environmental, cultural, and lifestyle factors. Indeed, our findings suggest that a comprehensive assessment of these interacting determinants may be more informative than focusing on genetic variability alone when investigating the complex relationship between taste perception and dietary behaviour.

An additional consideration is that participants were not systematically excluded based on metabolic, thyroid, hepatic, or renal disorders, pregnancy or lactation, or the use of medications potentially affecting taste or food preferences. This approach allowed the inclusion of a more heterogeneous community-based population, although the potential influence of these factors cannot be completely excluded and may represent a source of residual confounding. This study was designed to investigate interindividual variability in bitter taste perception and its potential relationship with food preferences and dietary behaviour in a general adult population, rather than to clinically assess disease-related taste alterations. Future studies may also consider a more comprehensive assessment of medical history and medication use.

Regarding participant recruitment, people were enrolled from companies, gyms, and universities. Although this strategy allowed for the recruitment of participants from different social and occupational settings, it may have resulted in some degree of selection towards younger or more health-conscious individuals. Therefore, caution should be exercised when generalising the findings to the broader Italian population.

Finally, anthropometric measurements and dietary information were self-reported and may consequently be subject to recall or social desirability bias. Despite these considerations, the multidimensional assessment adopted in the present study provides useful insights into the relationship between bitter taste perception, genetic variability, lifestyle factors, and dietary behaviour.

## 5. Conclusions

In conclusion, the present study confirmed a significant association between PROP sensitivity and the *TAS2R38* rs713598 genotype, supporting the role of this polymorphism in bitter taste perception. However, neither PROP sensitivity nor *TAS2R38* genotype was significantly associated with food preferences or adherence to the Mediterranean diet. These findings suggest that genetic variation in bitter taste perception alone may have a limited influence on broader dietary behaviour, which is likely shaped by a complex interplay of lifestyle, cultural, and experiential factors. Overall, our results highlight the multifactorial nature of dietary behaviour and support the importance of considering modifiable lifestyle factors alongside genetic and sensory determinants.

## Figures and Tables

**Table 1:** Smokers, weight condition, and physical activity evaluation of the sampled population.

	Male	Female	Total
	*n* (%)	*n* (%)	*n* (%)
**Smokers**	13 (20.0)	39 (29.1)	52 (26.1)
Cigarettes (*n*)/day ^a^			7.41 ± 5.78
**BMI**			
Underweight	1 (1.5)	8 (5.9)	9 (4.5)
Normal	45 (69.2)	108 (80.0)	153 (76.5)
Overweight	17 (26.2)	14 (10.4)	31 (15.5)
Obesity	2 (3.1)	5 (3.7)	7 (3.5)
**MET/min/week** ^a^	5078.15 ± 5181.78	2958.02 ± 4301.77	3647.06 ± 4699.71
Active	9 (13.8)	28 (20.7)	37 (18.5)
Sufficiently active	16 (24.6)	57 (42.2)	73 (36.5)
Inactive	40 (61.5)	50 (37.0)	90 (45.0)

^a^ Data expressed as the mean ± SD.

**Table 2:** Number and percentages of people who respect the indicated consumption frequency of the Mediterranean diet adherence questionnaire.

	Freq. ^a^	*n* (%) ^b^
1. Do you use extra-virgin olive oil as the primary source of fat for cooking?	Yes	194 (97.0)
2. How many servings of vegetables do you consume per day? (1 serving = 200 g)	≥2	118 (59.0)
3. How many fruits (including fresh-squeezed juice) do you consume daily?	≥3	49 (24.5)
4. How many servings of red meat, hamburgers, and sausages do you consume weekly? (1 serving = 100–150 g)	≤1	46 (23.0)
5. How many servings of butter, margarine, and cream do you consume weekly? (1 serving = 12 g)	<1	115 (57.5)
6. How many times a week do you consume sugar-sweetened and/or carbonated beverages?	<1	69 (34.5)
7. How many servings of pulses do you consume weekly? (1 serving = 150 g)	≥3	56 (28.0)
8. How many servings of fish and seafood do you consume weekly? (1 serving = 100–150 g of fish, 4–5 pieces or 200 g of seafood)	≥3	32 (16.0)
9. How many times a week do you consume pastry such as cookies, cake, or sweets?	<3	96 (48.0)
10. How many times a week do you consume nuts? (1 serving = 30 g)	≥3	71 (35.5)
11. Do you prefer chicken, turkey, or rabbit instead of beef, pork, hamburgers, or sausages?	Yes	97 (48.5)
12. Do you usually add sugar to drinks like coffee or tea?	No	120 (60.0)
13. How many servings of bread, pasta, and whole-wheat rice do you consume weekly? (1 serving = 75 g)	≥5	73 (36.5)
14. How many servings of bread, rice, and pasta with refined flour do you consume weekly? (1 serving = 75 g)	<3	75 (37.5)
15. How many times a week do you use sofrito (with oil, garlic, onion, etc.) to prepare meals?	≥2	115 (57.5)
16. On average, how many glasses of wine and/or beer and/or strong drinks do you consume weekly? (1 glass of wine = 125 mL; beer = 330 mL; aperitif = 80 mL; strong drink = 40 mL)	M 14–21 cups F 7–14 cups	6 (3.0)

^a^ Criteria to score 1 point. Otherwise, 0 is recorded; ^b^ Number and percentage of participants scoring 1 in the Mediterranean diet adherence questionnaire.

**Table 3:** Mediterranean diet (M.D.) adherence (er-MEDAS score) and Food Preference Score of the sampled population.

	Male	Female	Total
	*n* (%)	*n* (%)	*n* (%)
**Er-MEDAS score**	6.65 ± 0.28	6.67 ± 0.18	6.66 ± 0.15
M.D. low adherence	43 (66.2)	95 (70.4)	138 (69.0)
M.D. moderate/high adherence	22 (33.8)	40 (29.6)	62 (31.0)
**Food Preference Score**	87.42 ± 20.48	84.67 ± 20.94	85.56 ± 20.78

**Table 4:** PROP test score (bitter perception) and genotyping of the *TAS2R38* rs713598 SNP of the sampled population.

	Male	Female	Total	*E * ^b^	*χ* ^2^	*p*
	*n* (%)	*n* (%)	*n* (%)			
**Genotype**						
Super-taster (P/P)	9 (15.8)	28 (23.5)	37 (21.0)	37	0.0	1.0
Medium taster (P/A)	28 (49.1)	60 (50.4)	88 (50.0)	87	0.0	1.0
Non-taster (A/A)	20 (35.1)	31 (26.1)	51 (29.0)	51	0.0	1.0
**PROP test Score** ^a^	1.77 ± 2.12	2.51 ± 2.51	2.27 ± 2.41	/	/	/
Barely detectable—Weak	39 (60.0)	64 (47.4)	103 (51.5)	/	/	/
Moderate—Strong	22 (33.8)	48 (35.6)	70 (35.0)	/	/	/
Very strong—The strongest imaginable	4 (6.2)	23 (17.0)	27 (13.5)	/	/	/

^a^ Data expressed as the mean ± SD; ^b^ Expected genotype frequencies for the Hardy–Weinberg test: *f*(PAV) = 0.54; *f*(AVI) = 0.46; statistically significant with *p* < 0.05.

## Data Availability

The raw data supporting the conclusions of this article will be made available by the authors on request.

## Supplementary Materials

The following supporting information can be downloaded at: https://www.mdpi.com/article/10.3390/epidemiologia7050131/s1, Supplementary Figure S1: Foods and beverages included in the food preference questionnaire; Supplementary Figure S2: The Labelled Magnitude Scale adopted for the study; Supplementary Table S1. How unmodifiable factors (a), such as age, sex, bitter perception (PROP), and *TAS2R38* genotype with SNP rs713598, or modifiable (b) factors, namely smoking habit, weight condition (BMI), Mediterranean diet adherence (er-MEDAS), and physical activity (MET/min/week), influence Mediterranean diet adherence. The analysis was performed among participants with available genotyping data (*n* = 176). Supplementary Table S2. How unmodifiable factors (a), such as age, sex, bitter perception (PROP), and *TAS2R38* genotype with SNP rs713598, or modifiable (b) factors, namely smoking habit, weight condition (BMI), Mediterranean diet adherence (er-MEDAS), and physical activity (MET/min/week), influence food preferences. The analysis was performed among participants with available genotyping data (*n* = 176).

## Abbreviations

The following abbreviations are used in this manuscript:

TAS2R38	Taste 2 Receptor Member 38
BMI	Body Mass Index
DNA	Deoxyribonucleic Acid
Er-MEDAS	Energy-Restricted Mediterranean Diet Adherence Screener
SNP	Single Nucleotide Polymorphism
PTC	Phenylthiocarbamide
PROP	6-n-Propylthiouracil
PCR	Polymerase Chain Reaction
qPCR	Quantitative Polymerase Chain Reaction
LMS	Labelled Magnitude Scale
IPAQ	International Physical Activity Questionnaire
MET	Metabolic Equivalent of Task
PAV	Proline–Alanine–Valine
AVI	Alanine–Valine–Isoleucine
P/P	Proline/Proline
P/A	Proline/Alanine
A/A	Alanine/Alanine
SD	Standard Deviation
