# Supplementary material for: Correlation Between the TAS2R38 Genetic Polymorphism, PROP Perception and Eating Habits: A Cross-Sectional Molecular Epidemiological Study
Source: Epidemiologia (Basel). 2026 Sep 17;7(5):131. doi: 10.3390/epidemiologia7050131 (PMC13600190; doi:10.3390/epidemiologia7050131)
Supplement: Supplementary file 1 [file epidemiologia-07-00131-s001.zip › epidemiologia-4465385-supplementary.pdf]

SUPPLEMENTARY MATERIALS

Supplementary Figure S1. Foods and beverages included in the food preference questionnaire.

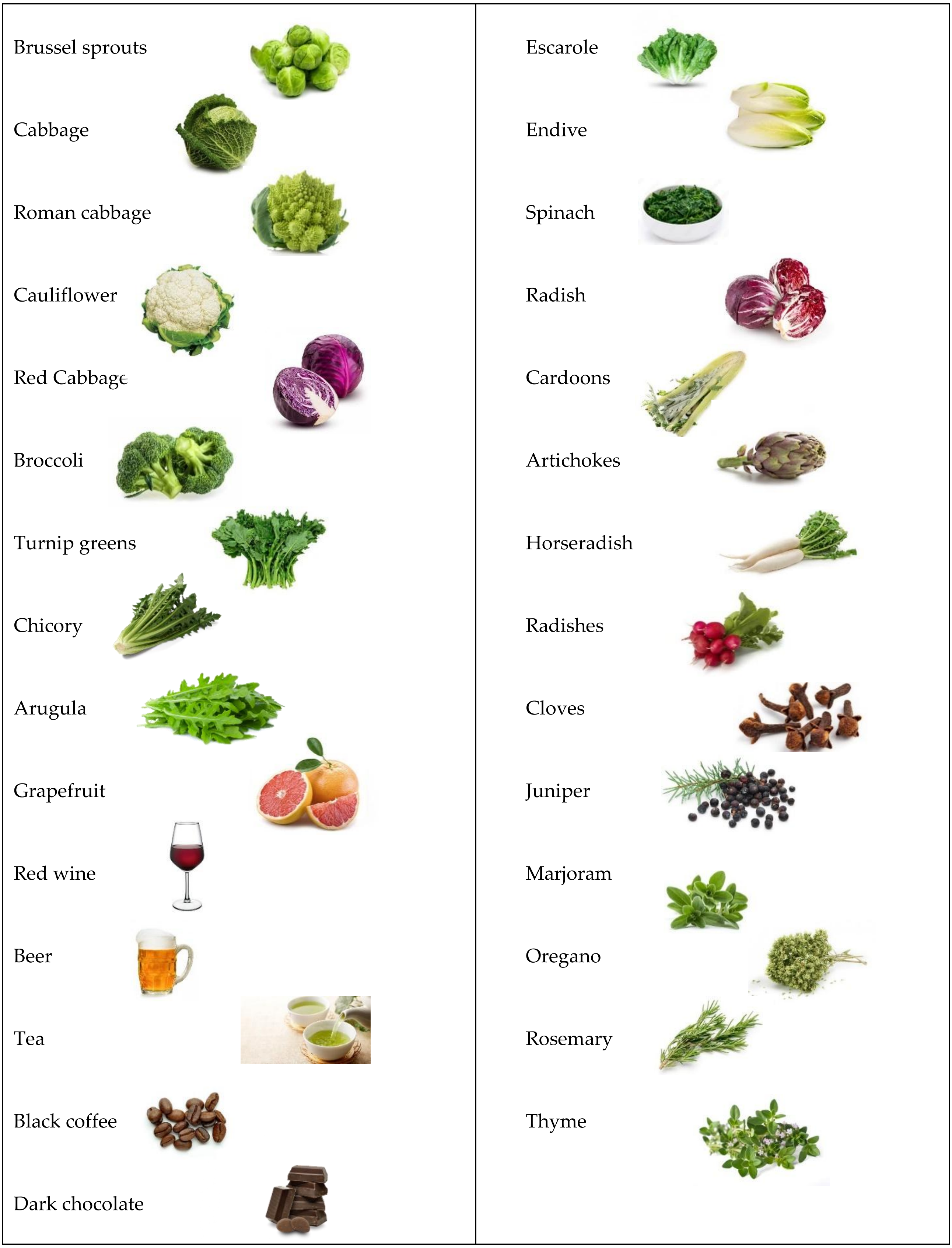

Supplementary Figure S2. The Labelled Magnitude Scale adopted for the study.

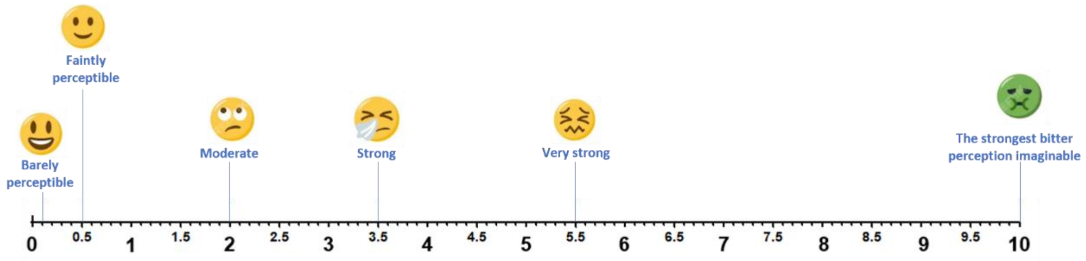

Supplementary Table S1. How unmodifiable factors (**a**), such as age, sex, bitter perception (PROP), and *TAS2R38* genotype with SNP rs713598, or modifiable (**b**) factors, namely smoking habit, weight condition (BMI), Mediterranean diet adherence (er-MEDAS), and physical activity (MET/min/week), influence Mediterranean diet adherence. The analysis was performed among participants with available genotyping data (n = 176).

(a)

	**Exp (B)**	**95% CI**	***p***
**Age**			
< 30 years	1.114	0.968 ± 1.282	0.113
≥ 30 years	1		
**Sex**			
Female	0.923	0.797 ± 1.068	0.282
Male	1		
**PROP test**			
Very strong – The strongest imaginable	0.998	0.806 ± 1.237	0.989
Moderate – Strong	0.471	0.802 ± 1.107	0.471
Barely detectable – Weak	1		
**Genotype**			
Super-taster (P/P)	0.986	0.798 ± 1.218	0.897
Medium-taster (P/A)	1.040	0.878 ± 1.231	0.651
Non-taster (A/A)	1		

(b)

	**Exp (B)**	**95% CI**	***p***
**Smokers**			
Yes	0.844 *	0.728 ± 0.978	0.024
No	1		
**BMI**			
Obesity	1.056	0.753 ± 1.482	0.751
Overweight	0.906	0.760 ± 1.082	0.276
Normal weight	1		
Underweight	1.055	0.751 ± 1.482	0.758
**MET/min/week**			
Active	1.237 *	1.035 ± 1.478	0.020
Sufficiently active	1.034	0.861 ± 1.242	0.719
Inactive	1		

* $p < 0.05$

Supplementary Table S2. How unmodifiable factors (**a**), such as age, sex, bitter perception (PROP), and *TAS2R38* genotype with SNP rs713598, or modifiable (**b**) factors, namely smoking habit, weight condition (BMI), Mediterranean diet adherence (er-MEDAS), and physical activity (MET/min/week), influence food preferences. The analysis was performed among participants with available genotyping data (n = 176).

(a)

	Exp (B)	95% CI	*p*
**Age**			
≥30 years	1.410 *	1.094 ± 1.817	0.008
<30 years	1		
**Sex**			
Female	0.772	0.592 ± 1.006	0.055
Male	1		
**PROP test**			
Very strong – The strongest imaginable	0.814	0.553 ± 1.198	0.296
Moderate – Strong	0.890	0.666 ± 1.190	0.431
Barely detectable – Weak	1		
**Genotype**			
Super-taster (P/P)	1.055	0.720 ± 1.546	0.782
Medium-taster (P/A)	1.083	0.798 ± 1.470	0.607
Non-taster (A/A)	1		

* $p < 0.05$

(b)

	Exp (B)	95% CI	*p*
**Smokers**			
Yes	0.844	0.639 ± 1.115	0.232
No	1		
**BMI**			
Obesity	0.826	0.436 ± 1.567	0.559
Overweight	0.899	0.644 ± 1.255	0.530
Normal	1		
Underweight	1.447	0.762 ± 2.751	0.259
**MET/min/week**			
Active	1.249	0.892 ± 1.749	0.196
Sufficiently active	0.976	0.690 ± 1.379	0.890
Inactive	1		
